# *Vital Signs:* Newly Reported Acute and Chronic Hepatitis C Cases ― United States, 2009–2018

**DOI:** 10.15585/mmwr.mm6914a2

**Published:** 2020-04-10

**Authors:** A. Blythe Ryerson, Sarah Schillie, Laurie K. Barker, Benjamin A. Kupronis, Carolyn Wester

**Affiliations:** 1 Division of Viral Hepatitis, National Center for HIV/AIDS, Viral Hepatitis, STD, and TB Prevention, CDC.

## Abstract

**Introduction:**

Hepatitis C is a leading cause of death from liver disease in the United States. Acute hepatitis C infection is often asymptomatic, and >50% of cases will progress to chronic infection, which can be life-threatening. Hepatitis C can be diagnosed with a blood test and is curable, yet new cases of this preventable disease are increasing.

**Methods:**

National Notifiable Diseases Surveillance System data were analyzed to determine the rate of acute hepatitis C cases reported to CDC by age group and year during 2009–2018 and the number and rate of newly reported chronic cases in 2018 by sex and age. The proportion of adults aged ≥20 years with hepatitis C who reported having ever been told that they had hepatitis C was estimated with 2015–2018 National Health and Nutrition Examination Survey data.

**Results:**

During 2018, a total of 3,621 cases of acute hepatitis C were reported, representing an estimated 50,300 cases (95% confidence interval [CI] = 39,800–171,600). The annual rate of reported acute hepatitis C cases per 100,000 population increased threefold, from 0.3 in 2009 to 1.2 in 2018, and was highest among persons aged 20–29 (3.1) and 30–39 years (2.6) in 2018. A bimodal distribution of newly reported chronic hepatitis C cases in 2018 was observed, with the highest proportions among persons aged 20–39 years and 50–69 years. Only 60.6% (95% CI = 46.1%–73.9%) of adults with hepatitis C reported having been told that they were infected.

**Conclusions and Implications for Public Health Practice:**

Increasing rates of acute hepatitis C among young adults, including reproductive-aged persons, have put multiple generations at risk for chronic hepatitis C. The number of newly reported chronic infections was approximately equal among younger and older adults in 2018. The new CDC hepatitis C testing recommendations advise screening all adults and pregnant women, not just persons born during 1945–1965, and those with risk factors.

## Introduction

Hepatitis C is a leading cause of morbidity and mortality from liver disease, costing the U.S. health care system billions of dollars annually (*1*,*2*). Hepatitis C virus (HCV) is primarily transmitted through direct percutaneous exposure to blood through injection drug use, but can also be transmitted sexually or from an infected mother to her infant during pregnancy or childbirth. HCV can cause an acute infection (acute hepatitis C), followed in some cases by chronic infection (chronic hepatitis C). Persons with acute hepatitis C are typically asymptomatic or have only a mild clinical illness. Acute hepatitis C infection might clear completely without any treatment, but >50% of infections will progress to chronic hepatitis C, which is also typically asymptomatic until liver damage is severe enough to cause symptoms (*3*,*4*). Left untreated, chronic hepatitis C can be life-threatening.

Despite availability of accurate diagnostic tests and highly effective curative treatment, approximately 2.4 million adults in the United States (i.e., approximately 1.0% of all U.S. adults) were living with hepatitis C during 2013–2016 (*5*). Because HCV infection is most often asymptomatic, only 55.6% of these adults reported having ever been told that they had hepatitis C during 2013–2016 (*6*). Being unaware of an HCV infection can have serious health consequences and increase risk for transmission to others. In 2018, ≥15,713 death certificates listed hepatitis C as the underlying or contributing cause of death (*7*).

Historically, the highest prevalence of chronic hepatitis C in the United States has been among persons born during 1945–1965 (baby boomers) (*8*). Concurrent with the nation’s opioid crisis, in more recent years, new HCV infections have occurred primarily among young adults, including persons of reproductive age. Compared with 2005 when reported acute hepatitis C cases were at a low point this century (0.2 per 100,000 population), by 2017 the rate had increased approximately fourfold, to 1.0 per 100,000, mostly among persons aged 20–39 years* (*9*). Further, a recent study of the Healthcare Cost and Utilization Project showed that U.S. rates of maternal HCV infection at delivery increased from 0.8 per 1,000 live births in 2000 to 4.1 in 2015, with the highest increases among women with opioid use disorder (*10*).

Because hepatitis C testing and curative treatment substantially reduces long-term risk for disease and death, in 2012 CDC augmented the risk-based testing guidelines to recommend screening all persons born during 1945–1965 (*8*). Because of the changing epidemiology of hepatitis C in the United States, CDC is now recommending screening of all adults at least once in their lifetime and screening of all pregnant women during every pregnancy (*11*). The purpose of this report is to highlight the epidemic of hepatitis C among all adults in support of this new CDC screening recommendation.

## Methods

National Notifiable Diseases Surveillance System (NNDSS) data for 2009–2018 were analyzed by age group to quantify the annual number of confirmed acute hepatitis C cases per 100,000 population (rate) reported to CDC. During 2016–2019, the Council of State and Territorial Epidemiologists (CSTE) defined a confirmed acute hepatitis C case as one that met both clinical and laboratory criteria, or test conversion criteria.^†^ To meet clinical criteria, a person must have been evaluated for discrete onset of any sign or symptom consistent with acute viral hepatitis and have had either jaundice or elevated serum alanine aminotransferase levels. To meet laboratory criteria, the person must have had a positive nucleic amplification test (NAT) for HCV RNA or HCV antigen test. Test conversion is defined as having received a positive anti-HCV test, HCV antigen test, or NAT within 12 months after a negative result for any of these tests. To account for underascertainment and underreporting in acute hepatitis C surveillance data, the total estimated number of cases of acute hepatitis C for 2018 was calculated using standard methodology as described in the CDC viral hepatitis annual surveillance reports (*9*,*12*).

To quantify the number and rate of confirmed chronic hepatitis C cases newly reported to CDC by sex, age, and social generation (i.e., birth cohort), NNDSS data for 2018 were analyzed. In 2018, CSTE defined a confirmed chronic hepatitis C case as one that did not meet the definition of an acute case and had a positive NAT for HCV RNA or HCV antigen test. A newly reported chronic case is a chronic hepatitis C case that meets the CSTE case definition and has not been reported previously.^§^ All acute and chronic hepatitis C rates were calculated using yearly U.S. population estimates from 2009–2018.^¶^ The p-values for all chi-squared tests were <0.001 and are not presented.

National Health and Nutrition Examination Survey (NHANES) data were analyzed to estimate the proportion of adults with confirmed current hepatitis C who reported having ever been told that they had hepatitis C. NHANES is an ongoing, nationally representative interview and examination survey of the U.S. noninstitutionalized population.** As part of the 2015–2016 and 2017–2018 surveys, participants were asked whether they had ever been told that they had hepatitis C and were then tested for hepatitis C during an examination (anti-HCV testing followed by reflex NAT testing for HCV RNA). Participants who answered “No” and had positive test results for HCV RNA were considered to be unaware of their infection. Those who refused to answer were classified as missing. Because the number of HCV RNA-positive NHANES participants during any given survey is small (e.g., 50 during 2015–2016 and 51 during 2017–2018), the two survey cycles were combined to improve stability of the estimates. The proportion of HCV RNA-positive persons aged ≥20 years aware of their infection during 2015–2018 was weighted to account for unequal probability of selection and nonresponse, and 95% Clopper-Pearson exact CIs accounted for the complex survey design. SUDAAN (version 11.0.1; RTI International) was used for the analyses. Stratifying the estimate by selected demographic and socioeconomic characteristics was attempted; however, no stratifications of interest met National Center for Health Statistics data presentation standards because of small cell counts (*13*).

## Results

**FIGURE 1:**
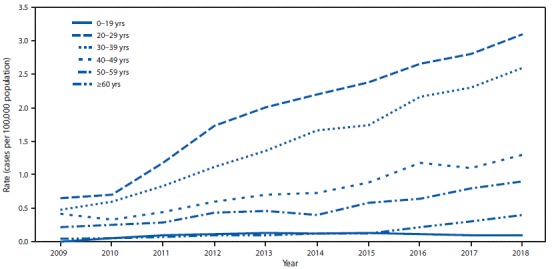
Rate* of reported^†^ acute hepatitis C cases,^§^ by year and age group — National Notifiable Diseases Surveillance System, United States, 2009–2018 * Cases per 100,000 U.S. population. ^^†^^ The states and jurisdictions reporting cases to CDC through the National Notifiable Diseases Surveillance System might vary by year (https://www.cdc.gov/hepatitis/statistics/2017surveillance/index.htm). During 2018, cases of acute hepatitis C were either not reportable by law, statute, or regulation; not reported; or otherwise unavailable to CDC from Alaska, Arizona, Delaware, District of Columbia, Hawaii, Iowa, Mississippi, and Rhode Island. ^^§^^ Only confirmed, acute hepatitis C cases are included. Complete case definitions by year are available at https://wwwn.cdc.gov/nndss/conditions/hepatitis-c-acute.

During 2018, a total of 3,621 cases of acute hepatitis C were reported, representing an estimated 50,300 cases (95% CI = 39,800–171,600), after adjusting for underascertainment and underreporting. During 2009–2018, the number of reported acute hepatitis C cases per 100,000 population increased threefold, from 0.3 in 2009 to 1.2 in 2018. During 2018, the highest rate of reported acute hepatitis C cases was in persons aged 20–29 years (3.1 per 100,000), followed by persons aged 30–39 years (2.6), 40–49 years (1.3), 50–59 years (0.9) and ≥60 years (0.4); the lowest rate (0.1) was in persons aged <20 years (Figure 1). This age pattern was consistent throughout 2009–2018, but the absolute increase in the annual case counts per 100,000 was highest for persons aged 20–39 years; among those aged 20–29 years, rates increased approximately 300%, from 0.7 in 2009 to 3.1 in 2018, and among those aged 30–39 years, rates increased approximately 400%, from 0.5 in 2009 to 2.6 in 2018.

**FIGURE 2:**
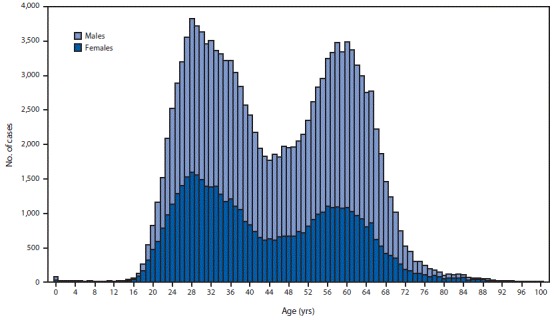
Number of newly reported* chronic hepatitis C cases,^†^ by sex and age — National Notifiable Diseases Surveillance System, United States, 2018 * During 2018, cases of chronic hepatitis C were either not reportable by law, statute, or regulation; not reported; or otherwise unavailable to CDC from Alabama, Arizona, Arkansas, California, Delaware, District of Columbia, Hawaii, Indiana, Kentucky, Mississippi, Nevada, North Carolina, Rhode Island, and Texas. ^^†^^ Only confirmed, newly diagnosed, chronic hepatitis C cases with information regarding both sex and age are included. Complete case definition is available at https://wwwn.cdc.gov/nndss/conditions/hepatitis-c-chronic/case-definition/2016/.

A total of 137,713 new chronic hepatitis C cases were reported during 2018. A larger percentage of these cases were among males (63.1%) than among females (36.9%) (Table). Among both males and females, a bimodal age distribution was observed, with the largest proportion of all newly reported chronic cases among persons aged 20–39 and 50–69 years (Figure 2). Baby boomers accounted for 36.3% of newly reported chronic hepatitis C cases in 2018, persons born during 1966–1980 (Generation X) accounted for 23.1%, and those born during 1981–1996 (millennials) accounted for 36.5%. (Table). Among 2015–2018 NHANES participants aged ≥20 years who were HCV RNA-positive, 60.6% (95% CI = 46.1%–73.9%) reported having been told that they had hepatitis C.

**TABLE:** Newly reported* chronic hepatitis C cases,^†^ by characteristic — National Notifiable Diseases Surveillance System, United States, 2018

Characteristic	No. (%)	Rate^§^
**Total**	**137,713 (100.0)**	**54.1**
**Sex**		
Male	86,670 (63.1)	69.1
Female	50,730 (36.9)	39.2
**Age group (yrs)**		
0–19	1,302 (0.9)	2.1
20–29	25,353 (18.4)	72.0
30–39	32,223 (23.4)	95.0
40–49	19,707 (14.3)	62.8
50–59	28,385 (20.7)	84.1
60–69	25,360 (18.5)	85.8
≥70	5,104 (3.7)	18.2
**Social generation (birth cohort)^¶^**		
Alpha (born after 2012)	176 (0.1)	1.0
Generation Z (born 1997–2012)	3,120 (2.3)	6.1
Millennial (born 1981–1996)	50,160 (36.5)	89.7
Generation X (born 1966–1980)	31,688 (23.1)	66.7
Baby boomers (born 1945–1965)	49,940 (36.3)	79.8
Silent (born 1928–1944)	2,246 (1.6)	—**
Greatest (born 1901–1927)	104 (0.1)	—**

* During 2018, cases of chronic hepatitis C were either not reportable by law, statute, or regulation; not reported; or otherwise unavailable to CDC from Alabama, Arizona, Arkansas, California, Delaware, District of Columbia, Hawaii, Indiana, Kentucky, Mississippi, Nevada, North Carolina, Rhode Island, and Texas.

^†^ Only confirmed, newly diagnosed, chronic hepatitis C cases are included. Complete case definition is available at https://wwwn.cdc.gov/nndss/conditions/hepatitis-c-chronic/case-definition/2016/.

^§^ Cases per 100,000 population.

**^¶^** In 2018, persons categorized in the Alpha Generation (born since 2012) were aged 0–5 years, Generation Z (born 1997–2012) were aged 6–21 years, millennials (born 1981–1996) were aged 22–37 years, Generation X (born 1966–1980) were aged 38–52 years, baby boomers (born 1945–1965) were aged 53–73 years, the Silent Generation (born 1928–1944) were aged 74–90 years, and the Greatest Generation (born 1901–1927) were aged ≥91 years.

** Rates cannot be calculated because single-year population size for persons aged ≥85 years are not available.

## Discussion

Historically, CDC has focused hepatitis C screening efforts among persons born during 1945–1965 and testing among those with identified risk factors regardless of age (*8*,*14*,*15*). Concurrent with the nation’s opioid crisis, however, rapid increases in acute HCV infections among young adults, including reproductive-aged persons, have put multiple U.S. generations at risk for chronic HCV infection. In today’s issue of *MMWR Recommendations and Reports*, CDC recommends a universal testing strategy for hepatitis C among adults, including pregnant women (*11*).

CDC first began publishing recommendations for hepatitis C screening in 1991, when the U.S. Public Health Service (USPHS) issued guidelines recommending hepatitis C testing of all blood and organ donations intended for human use (*16*). In 1998, CDC expanded the hepatitis C interagency testing guidelines to include a recommendation for testing persons at high risk. These persons were defined as those who had ever injected drugs and shared needles, syringes, or other drug preparation equipment; received clotting factor concentrates produced before 1987; had ever been on maintenance hemodialysis; had persistently abnormal alanine aminotransferase levels; received blood transfusions or organ transplants before July 1992; had a recognized exposure (e.g., a needlestick or other sharps injury); or were born to a mother infected with HCV (*14*). In 1999, USPHS added persons with human immunodeficiency virus to the groups recommended for testing (*15*). In 2012, because of concern regarding limited effectiveness of risk-based hepatitis C testing and a high prevalence of disease among persons born during 1945–1965, CDC augmented the risk-based testing guidelines with a recommendation for a one-time testing of all baby boomers, even in the absence of a known risk factor (*8*).

Ecologic evidence reveals that CDC’s 2012 recommendation to screen all baby boomers for HCV infection resulted in increased testing among that birth cohort (*17*). However, existing testing strategies have had limited success because >39% of all adults with HCV infection still report being unaware that they are infected. Further, the increase in new acute and newly reported chronic infections among young adults further supports the need for expanded and easier-to-implement hepatitis C testing recommendations. The new CDC recommendations released today include screening of all adults aged ≥18 years once in their lifetime and screening of all pregnant women during each pregnancy (*11*). They also include an exception for settings where the prevalence of HCV infection is <0.1%; however, there are few known settings with a hepatitis C prevalence below that threshold (*18*,*19*). The recommendation for testing of persons with risk factors remains in effect, regardless of age or setting prevalence, including continued periodic testing of persons with ongoing risk. The U.S. Preventive Services Task Force (USPSTF) recently published a recommendation statement on screening for hepatitis C virus infection in all adults aged 18–79 years (B recommendation) (*20*). The USPSTF recommendation differs from CDC’s recommendation in that 1) an upper age limit is defined, 2) there is no recommendation for screening during every pregnancy, 3) and a prevalence threshold at which universal screening would remain cost-effective is not identified.

The findings in this report are subject to at least three limitations. First, the number of cases of acute and chronic hepatitis C reported to CDC underestimate the actual incidence of disease, and not all states reported chronic infections to CDC in 2018. For every reported case of acute hepatitis C, CDC estimates that there are 13.9 actual cases (*9*,*12*); however, this estimation methodology is imprecise and might be influenced by testing rates. Second, minor changes to the CSTE case definition, changes to the reporting practices across jurisdictions, and changes to hepatitis C testing practices among providers during 2009–2018 should be considered when examining acute hepatitis C cases temporally. Finally, because NHANES sampling is limited to the noninstitutionalized, civilian population, survey results related to hepatitis C might not be nationally representative because they do not include some populations at highest risk for hepatitis C (e.g., incarcerated persons).

These findings highlight the need for immediate implementation of the new CDC universal hepatitis C screening recommendations for all adults and pregnant women (*11*). Following a decade of sharp increases in acute hepatitis C infections, particularly among young adults, the rates of newly reported chronic infections among baby boomers and millennials are now equal, demonstrating that even younger generations are at risk. Diagnosing HCV infection is a necessary first step to linking persons to cure to prevent life-threatening consequences of long-term chronic infections and transmission to others.

**Summary**

**What is already known about this topic?**

Acute hepatitis C infection is often asymptomatic, but >50% of cases will progress to chronic infection, which can be life-threatening. Hepatitis C can be diagnosed with a blood test and is curable.

**What is added by this report?**

The annual rate of reported acute hepatitis C tripled from 2009 to 2018 and was highest among persons aged 20–39 years. In 2018, the largest proportion of chronic hepatitis C cases occurred among persons aged 20–39 years and 50–69 years. Only 61% of adults with hepatitis C knew that they were infected.

**What are the implications for public health practice?**

All adults and pregnant women should be screened for hepatitis C according to CDC’s new screening recommendations.

## Footnotes

* Historical CDC viral hepatitis surveillance data are available at https://www.cdc.gov/hepatitis/statistics/SurveillanceRpts.htm.

† Complete CSTE case definitions for confirmed acute hepatitis C for 2011, 2012, and 2016 are available at https://wwwn.cdc.gov/nndss/conditions/hepatitis-c-acute.

§ The 2016 CSTE definition for confirmed chronic hepatitis C is available at https://wwwn.cdc.gov/nndss/conditions/hepatitis-c-chronic/case-definition/2016.

¶ U.S. population estimates were calculated using the Vintage 2018 Bridge-Race Postcensal Population Estimates available at https://www.cdc.gov/nchs/nvss/bridged_race.htm with states excluded as determined by case reporting status of acute hepatitis C.

** https://www.cdc.gov/nchs/nhanes/index.htm.
